# Analysis of treatment of large abdominal malignancies in children complicated with abdominal compartment syndrome: Report of six cases

**DOI:** 10.1097/MD.0000000000006705

**Published:** 2017-04-28

**Authors:** Zeng Le-Xiang, Wu Yao-Hao, Li Na, Qiu Rong-Lin, Zhou Jia-Jia, Jiang Wen-Li, Zhang Jie, Deng Xiao-Geng

**Affiliations:** a Department of Pediatric Surgery; b Department of Endocrinology, Sun Yat-sen Memorial Hospital, Guangdong Provincial Key Laboratory of Malignant Tumor Epigenetics and Gene Regulation Medical Research Center, Sun Yat-sen University, Guangzhou, China.

**Keywords:** abdominal compartment syndrome, abdominal malignancy, children, decompression, emergency operation, intra-abdominal pressure

## Abstract

To explore effective treatment of large abdominal malignancies in children complicated with abdominal compartment syndrome (ACS).

Six children with large abdominal malignancies complicated with ACS were admitted to our department from January 2013 to January 2016, and the changes in their breathing, heart rate, oxygen saturation, abdominal circumference, bladder pressure, and urine output, as well as the treatment measures and outcomes, were retrospectively analyzed.

The 6 children included 1 child with bilateral nephroblastoma, 1 child with abdominal alveolar rhabdomyosarcoma, 1 child with right ovarian malignant teratoma complicated with abdominal glioma, 1 child with abdominal malignant teratoma, 1 child with right nephroblastoma, and 1 child with left adrenal gland neuroblastoma. All patients were treated in a timely manner. The first 4 children underwent abdominal cavity decompression through surgical resection of the tumor, and the ACS was successfully cured allowing for follow-up care, whereas the last 2 patients failed to receive emergency surgery and eventually died due to the gradual aggravation of ACS.

Decompression through surgical resection of the tumor is the only effective measure for treating large abdominal malignancies in children complicated with ACS.

## 1 Introduction

Abdominal compartment syndrome (ACS) refers to the systematic dysfunction of multiple organs, including the heart and blood vessels, lung, kidney, stomach and intestine, and the brain, resulting from a marked increase in intra-abdominal pressure (IAP) from any cause.^[1–3]^ During ACS, the IAP is sustained at more than 20 mm Hg. Clinical risk factors for the increase in IAP include extensive burns, massive rehydration, pancreatitis, mechanical intestinal obstruction, post-abdominal-closure under tension, postoperative bleeding, retroperitoneal hemorrhage, and abdominal tumor. In particular, children with large abdominal malignancies show no obvious early symptoms, and most are unable to complain about the physical discomfort. However, the tumors are generally large when diagnosed and developed rapidly, leading to a sharp increase in IAP that is prone to be complicated with ACS. Clinically, the condition is a rare emergency situation that has a high mortality rate. In the past 3 years, 6 children with large abdominal malignancies complicated with ACS were admitted to our department, and the details of their cases were analyzed and reported in this paper.

## 2 Methods

### 2.1 Patients

From January 2013 to January 2016, 6 children suffering from large abdominal malignancies complicated with ACS were admitted to our department, including 3 males and 3 females aged 1 month to 3 years old with a mean age of 19 months old. The children weighed 5 to 13.5 kg, with a mean of 10.5 kg. The 6 children included 1 child with bilateral nephroblastoma, 1 child with abdominal alveolar rhabdomyosarcoma, 1 child with right ovarian malignant teratoma complicated with abdominal glioma, 1 child with abdominal malignant teratoma, 1 child with right nephroblastoma, and 1 child with left adrenal gland neuroblastoma. All children showed symptoms and signs including shortness of breath, obvious abdominal distension, and fast heart rate upon admission.

### 2.2 Treatment measures

After admission, the children were immediately given life support such as oxygen and fluid resuscitation, and vital signs and blood oxygen saturation were monitored. A urinary catheter was placed, urine output was monitored, and bladder pressure was measured. The presence of respiratory, circulatory, and urinary system dysfunction was assessed, and corresponding symptomatic treatment was started. Once ACS was diagnosed, abdominal decompression was immediately performed using surgical methods based on the specific circumstances of the child. A longitudinal or transverse incision of the abdominal wall was created, then the tumor was removed, and staged closure of the abdominal wall was performed.

### 2.3 IAP determination

In our department, intravesical pressure was measured to indirectly determine the IAP. The specific methods were as follows. The children were placed in the supine position with a Foley catheter inserted to empty the bladder. A transparent extension tube was added and connected to a 3-way connector. The catheter and the extension tubes were lifted to inject saline into the bladder with injection volumes of 1, 2, and 3 mL/kg with a maximum volume of 50 mL. The midaxillary line was used as the baseline; the vertical distance between the baseline and the lowest point of the liquid level was measured with a scale, and the values were recorded when no significant difference was present between the 2 measured values. These values were converted into IAP measurements according to the formula, 1 cm H_2_O = 0.74 mm Hg.^[4,5]^

### 2.4 Clinical observation indicators

The bladder pressure, respiration, heart rate, oxygen saturation, and urine output of the 4 children who underwent surgery were collected 6 hours before and 12 hours after surgery for analysis, and the abdominal circumference was compared before and after surgery.

## 3 Results

All of the 6 children suffered from large abdominal malignancies that had progressed after admission. These conditions were diagnosed as large abdominal malignancies complicated with ACS.

### 3.1 Case of bilateral nephroblastoma

**Figure 1:**
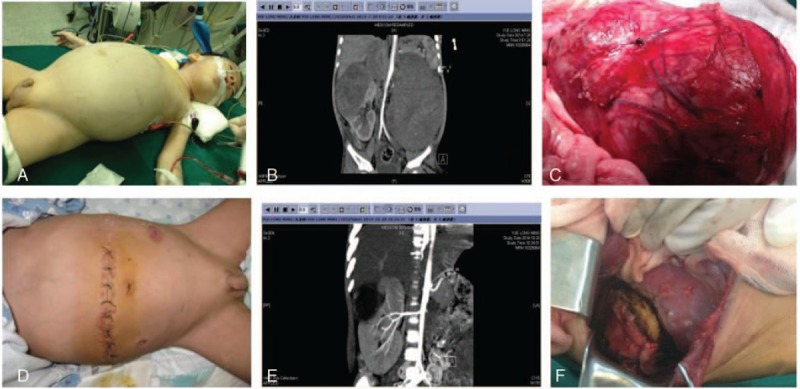
Case of bilateral nephroblastoma, A, Abdominal circumference of 53 cm, IAP of 25 mm Hg, and blood oxygen saturation falling to 80%. B, Almost no normal renal tissue was visible on the left side. The right tumor occupied more than half of the right kidney. C, The left kidney tumor seen at the first operation. Note large volume and rich blood supply. D, After the first operation abdominal circumference of 42 cm, IAP of 5 mm Hg, stable vital signs. E, Compensatory growth of the right kidney after resection of the left kidney. The tumor was occupying more than 1/4 of the right kidney. F, Partial resection of the right kidney in the second surgery, with the normal renal tissue remained. IAP = intra-abdominal pressure.

The first child suffered from bilateral nephroblastoma. After admission, a bilateral tumor biopsy was performed under ultrasound guidance, and neoadjuvant chemotherapy was planned after obtaining the pathological results. However, during the waiting process, the child showed gradually aggravated abdominal distension, poor mental reaction, shortness of breath, a rapid pulse, a decrease in blood oxygen saturation to 80%, and oliguria, with an abdominal circumference of 53 cm and IAP of 25 mm Hg. Because conservative treatment achieved poor results, resulting in a prolongation of the critical condition, surgery was immediately performed to remove the left nephroblastoma, followed by right renal tumor biopsy. After 2 courses of postoperative chemotherapy, the right renal tumor shrank, followed by a compensatory increase of the normal kidney tissue. Then, radical resection of the right nephroblastoma (partial resection of the right kidney) was performed, with good recovery. After surgery, the abdominal circumference was 42 cm, and the IAP was 5 mm Hg. Follow-up chemotherapy was initiated (Fig. 1).

### 3.2 Case of abdominal embryonal rhabdomyosarcoma

**Figure 2:**
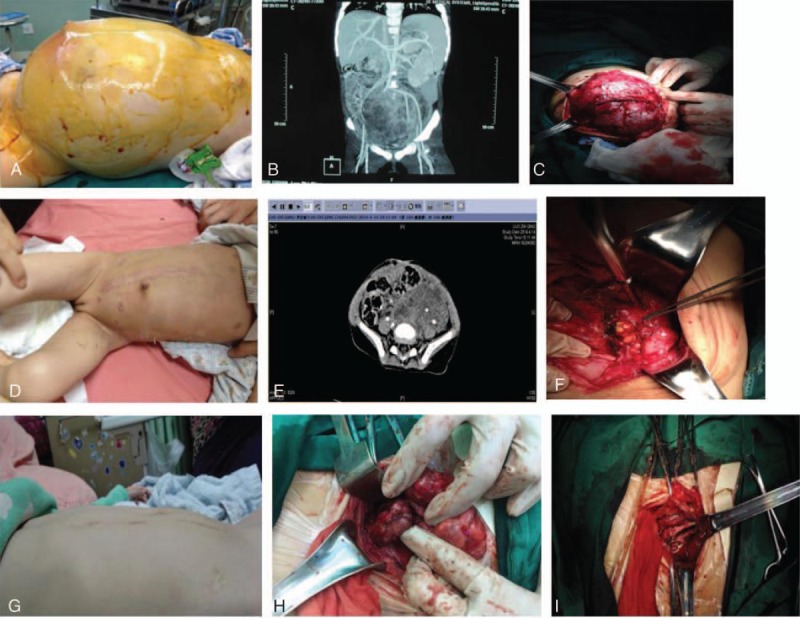
Case of abdominal enbryonal rhabdomyosarcoma. A, The clinical condition of the child deteriorated at the first day of chemotherapy. Blood oxygen saturation declined to 75%, abdominal circumference increased to 65 cm, and IAP increased to 30 mm Hg. B, CT scan obtained before the first operation showed tumor occupying almost the entire abdominal cavity, surrounding the left iliac vessels and the left ureter. C, The tumor showed massive blood supply. Operative reduction of tumor size was performed. D, After the first operation abdominal circumference declined to 43 cm, IAP declined to 8 mm Hg, and vital signs improved. E, CT before the second surgery suggested significantly decreased tumor size. F, After the second surgical resection of the tumor the pathologic examination confirmed necrosis of the tumor, and no vital tumor cells were observed. G, One year after the first operation recurrence of the tumor was noted. H, In the third operation, the recurrent tumor was noted to surround the left external iliac artery and vein. I, After transecting the left external iliac vein, the tumor was removed. Vascular anastomosis was performed. CT = computed tomography, IAP = intra-abdominal pressure.

In this child suffering from abdominal embryonal rhabdomyosarcoma, neoadjuvant chemotherapy was planned first. On the day we started chemotherapy, the condition of the child deteriorated, the breathing and heart rate increased significantly, and the blood oxygen saturation decreased to 75%, with an abdominal circumference of 65 cm and IAP of 30 mm Hg. Because the condition was life-threatening, surgery was immediately performed, and the intraoperative findings showed that the tumor occupied almost the entire abdominal cavity, wrapping around the left iliac blood vessels and left ureter. Therefore, atypical resection (leaving part of the tumor in place) of the large abdominal rhabdomyosarcoma was performed, combined with left ureter anastomosis and cystostomy. After surgery, the IAP decreased to 8 mm Hg, the abdominal circumference was reduced to 43 cm, and vital signs could be maintained. Regular chemotherapy was conducted for 11 weeks after surgery, and then a secondary surgery was performed. Retroperitoneal rhabdomyosarcoma resection and abdominal wall tumor resection, followed by regular chemotherapy, with no radiation therapy was used. One year and 10 months after surgery, tumor recurrence was found, and the operation was performed again. The intraoperative findings showed that the tumor surrounded the left external iliac artery and vein. After the left external iliac vein was truncated, the tumor was completely removed, and then anastomosis of the left external iliac vein was performed (Fig. 2).

### 3.3 Case of right ovarian malignant teratoma complicated by gliomatosis peritonei

**Figure 3:**
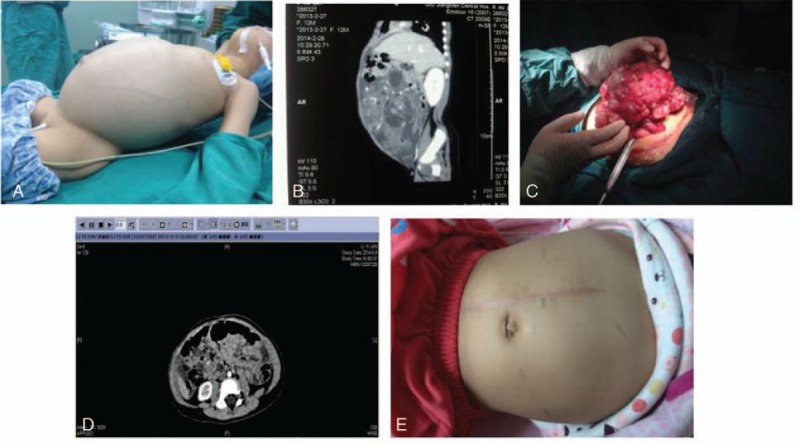
Child suffering from malignant teratoma of the right ovary complicated by gliomatosis peritonei. A, Preoperatively, blood oxygen saturation had declined to 88%, abdominal circumference had increased to 60 cm, and IAP had increased to 26 mm Hg. B, CT suggested large abdominal tumor, with massive abdominal effusion, right pleural effusion, pericardial effusion, alpha-1-fetoprotein: 380.70 ng/mL. C, Big malignant tumor of the right ovary complicated by gliomatosis peritonei of the abdominal wall and omentum. D, CT scan obtained postoperatively confirmed absence of residual tumor. E, Postoperative blood oxygen saturation and IAP returned to normal values, abdominal circumference of 41 cm, alpha-1-fetoprotein: 1.58 ng/mL.

One child had a right ovarian malignant teratoma complicated by gliomatosis peritonei. Before admission, computed tomography at another hospital suggested massive abdominal effusion, right pleural effusion, and pericardial effusion. After admission, an abdominal tumor biopsy was performed under ultrasonographic guidance. Alpha-1-fetoprotein was 380.7 ng/mL. Then, the child showed progressive abdominal distension, impairment of respiration, poor appetite, oliguria, and the blood oxygen saturation decreased to 88%, with an abdominal circumference of 60 cm and IAP of 26 mm Hg. The condition was diagnosed as ACS. Surgery was performed to remove the right ovarian large teratoma, omentum, and right subphrenic tumor. After surgery, the blood oxygen saturation and IAP returned to normal levels, with an abdominal circumference of 41 cm. Alpha-1-fetoprotein was 1.5 ng/mL. The clinical condition of the child improved, and we were able to start chemotherapy (Fig. 3).

### 3.4 Case of large abdominal tumor in an infant aged 1 month

**Figure 4:**
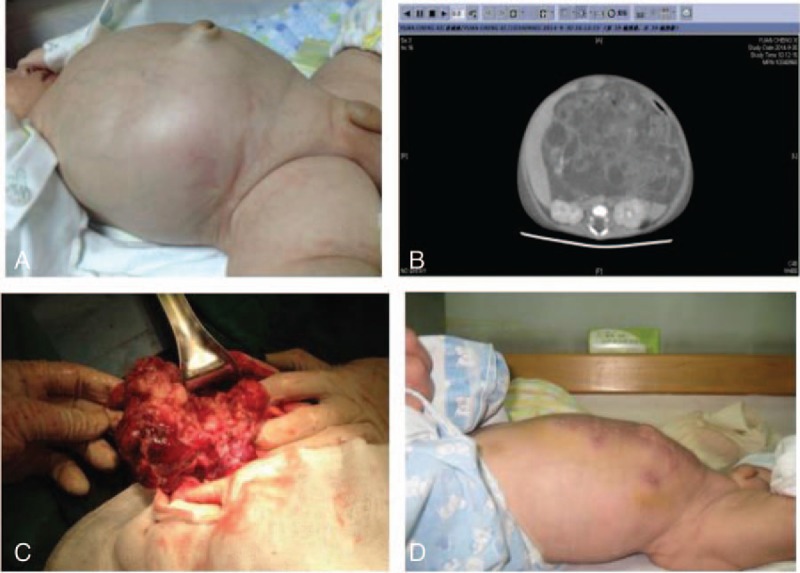
Case of abdominal malignant teratoma. A, Shortness of breath, blood oxygen saturation of 93% under administration of supplementary oxygen, oliguria, abdominal circumference of 48 cm, and IAP of 27 mm Hg. B, CT scan obtained preoperatively showed tumor, occupying almost the entire abdominal cavity. C, Resection of large abdominal tumor and abdominal decompression, postoperative pathologic investigation confirmed the diagnosis of an immature teratoma. D, Postoperatively, the abdominal circumference decreased to 37 cm, and IAP declined to 7 mm Hg.

After admission, the infant showed slowed psychomotor response, low fever, shortness of breath, blood oxygen saturation fluctuating between 85% and 93% under supplementary oxygen, and oliguria, with an abdominal circumference of 48 cm and IAP of 27 mm Hg. Since the condition of the infant deteriorated progressively, surgery was performed to remove the large abdominal tumor. The pathological diagnosis after surgery suggested immature teratoma. The abdominal circumference measured 37 cm, IAP was 7 mm Hg, and we were able to initiate chemotherapy (Fig. 4).

### 3.5 The final two cases of right nephroblastoma and left adrenal gland neuroblastoma

The remaining 2 children suffered from right-sided nephroblastoma and left adrenal neuroblastoma with progressively increased IAP and gradually aggravated symptoms. The families of the 2 children refused to accept surgical treatment, and thus the patients did not undergo abdominal decompression and eventually died. The details are shown in Table 1.

**Table 1:**
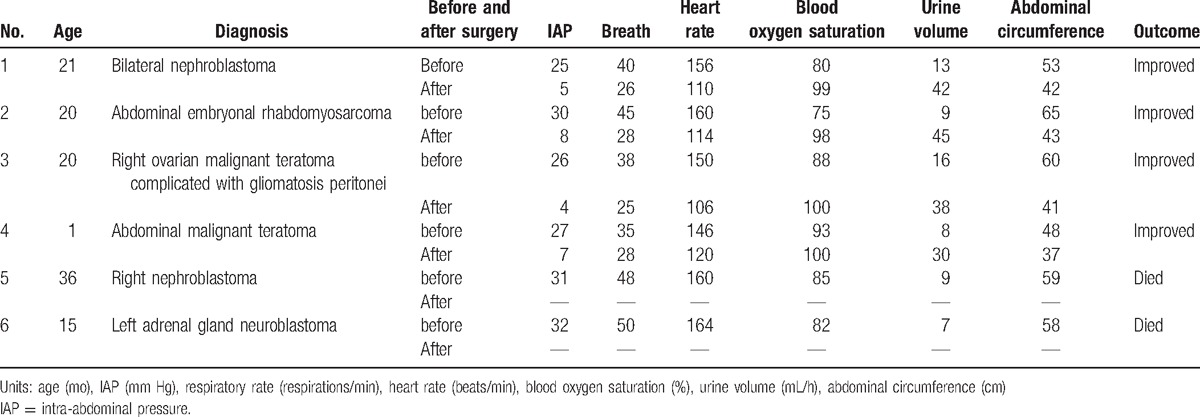
Parameter changes and outcomes in the 6 patients before and after operation.

## 4 Discussion

### 4.1 Clinical characteristic of ACS

The typical manifestation of ACS is abdominal distension, which presents as rapidly progressing abdominal distension accompanied by increasing respiratory resistance, progressive decline of blood oxygen saturation, oliguria or anuria, as well as multiple organ dysfunctions, such as azotemia, respiratory failure, decreased intestinal and hepatic blood flow, and low cardiac output syndrome. The mortality from the disease is high.^[6]^ Upon the onset of ACS, effective abdominal decompression is urgently required. The commonly used direct decompression measures, such as gastrointestinal decompression, abdominal puncture drainage decompression, and laparotomy decompression, can often alleviate the condition and effectively reverse the organ dysfunction, thereby reducing mortality.^[7,8]^ However, as abdominal malignancies in children are mostly composed of solid components, gastrointestinal decompression and abdominal puncture drainage decompression can be ineffective. Clinically, an IAP > 25 mm Hg is often used as an indication for decompressive laparotomy,^[9]^ but we believe that pediatric cases should not fully follow the adult grading standards, and the surgical indications can be extended according to the children's specific circumstances. In particular, for children suffering from large abdominal tumors complicated by ACS, laparotomy decompression is the only effective treatment. Hence, our indications for laparotomy decompression should be based on the pathophysiological changes instead of the measured values.^[6]^

### 4.2 Clinical characteristics of and therapeutic methods for large abdominal malignancies in children

Large abdominal malignancies in children are frequently characterized by the following parameters: rapid progression, generally large tumor upon diagnosis, rich blood supply to the tumor, and tumor tissue surrounding vital organs or blood vessels. As a result, heavy bleeding that is difficult to control often occurs intraoperatively, and the tumor cannot be completely removed through surgery. During the resection of large tumors, life-threatening incidents may occur. In addition, the previous consensus for treating large abdominal malignancies in children was to conduct neoadjuvant chemotherapy first and then to perform surgical resection of the tumor after the tumor volume had decreased. Because of the above limitations, controversy still exists regarding the priority of chemotherapy or surgical decompression for children with large abdominal malignancies.^[10]^ The majority of authors recommend using the following approach for treatment (provide literature): if the tumor is not complicated by ACS, neoadjuvant chemotherapy is started first, and then surgical resection is performed after the tumor volume has decreased. If the tumor is complicated by abdominal compartment syndrome, surgical abdominal decompression is conducted immediately. This strategy prevents further compression of the inferior vena cava and the portal vein.^[11]^ During chemotherapy, tumor necrosis or intratumoral hemorrhage resulting in increased volume of the tumor in the short term can also aggravate the process of ACS. For the second case in this paper, a child who suffered from abdominal alveolar rhabdomyosarcoma complicated with ACS, neoadjuvant chemotherapy was performed first, but on the day that chemotherapy was started, abdominal distension worsened, and the blood oxygen saturation deteriorated. Therefore, laparotomy decompression was immediately performed, and the presence of tumor necrosis and intratumoral hemorrhage was confirmed intraoperatively. The families of our fifth and sixth patients refused to accept surgical treatment, and ACS continued to intensify, causing irreversible dysfunction of multiple organs and ultimately leading to death. Therefore, for large abdominal malignancies in children complicated with ACS, after obtaining the consent of the patient's family, laparotomy decompression should be performed immediately.

### 4.3 Our treatment experience with large abdominal malignancies in children complicated with ACS

Because great risks exist for surgery for large abdominal malignancies in children complicated with ACS, the primary purpose of abdominal decompression is to reduce the abdominal pressure to save the child's life. Although a complete resection of the tumor through surgery is ideal, we believe that the complete removal of the tumor should not be blindly pursued. In this paper, for the first case of bilateral nephroblastoma complicated with ACS and the second case of abdominal alveolar rhabdomyosarcoma complicated with ACS, both had undergone 2 and 3 previous operations before the decompressive operation was necessary. Therefore, the first surgery was intended to remove a big part of tumor, using the irregular resection approach to effectively reverse the multiple organ dysfunction caused by ACS. After abdominal decompression, the children could undergo chemotherapy in a stable state, and the residual tumor shrank after chemotherapy, further providing conditions for a complete resection of the tumor. In the case of ACS occurring after the decompressive laparotomy, the delayed abdominal closure strategy is usually adopted.^[12]^ For this reason, we adopted abdominal effusion for drainage and closed the abdominal wall primarily. This surgical strategy prevented postoperative bleeding that would be difficult to control.

## 5 Conclusions

In summary, abdominal decompression with surgical resection of the tumor is effective, and may be the only effective measure, to save children with large abdominal malignancies complicated by ACS.

## Acknowledgments

This study was supported by the Province Special Basic and Applied Basic Research Program (Guangdong Province Natural Free Application Programs 2015A030313037),

The Guangzhou Collaborative Innovation of Industry, and the Major Projects of the People's Livelihood Project of Science and Technology (201604020151). This work was supported by the Key Laboratory of Malignant Tumor Molecular Mechanisms and Translational Medicine of Guangzhou Bureau of Science and Information Technology and the Key Laboratory of Malignant Tumor Gene Regulation and Target Therapy of Guangdong Higher Education Institutes.
